# Human in vitro-induced regulatory T cells display Dlgh1 dependent and PKC-θ restrained suppressive activity

**DOI:** 10.1038/s41598-017-04053-5

**Published:** 2017-06-26

**Authors:** Alexandra Zanin-Zhorov, Sudha Kumari, Keli L. Hippen, Sarah C. Merkel, Margaret L. MacMillan, Bruce R. Blazar, Michael L. Dustin

**Affiliations:** 1 Molecular Pathogenesis Program, Skirball Institute of Biomolecular Medicine, Department of Pathology, New York University School of Medicine, New York, NY 10016 USA; 2 University of Minnesota Cancer Center and Department of Pediatrics, Division of Blood and Marrow Transplantation, Minneapolis, MN 55455 USA; 3 Kadmon Corporation, LLC, New York, NY 10016 USA; 4 Koch institute of Integrative Cancer Research, MIT, Cambridge, MA-02139 USA; 5 Kennedy Institute of Rheumatology, University of Oxford, Oxford, OX3 7FY UK

**Keywords:** T cells, Signal transduction

## Abstract

*In vitro* induced human regulatory T cells (iTregs) have demonstrated *in vivo* therapeutic utility, but pathways regulating their function have not been elucidated. Here, we report that human iTregs generated *in vitro* from naïve cord blood cells preferentially recruit Disc large homolog 1 (Dlgh1) and exclude protein kinase C (PKC)-θ from immunological synapses formed on supported lipid bilayers with laterally mobile ICAM-1 and anti-CD3 mAb. Also, iTregs display elevated Dlgh1 overall and Dlgh1-dependent p38 phosphorylation, higher levels of phosphatase and tensin homolog (PTEN), and diminished Akt phosphorylation. Pharmacological interruption of PKC-θ increases and Dlgh1 silencing decreases the ability of iTregs to suppress interferon-γ production by CD4^+^CD25^−^ effector T cells (Teff). Comparison with expanded cord blood-derived CD4^+^CD25^hi^ tTreg and expanded Teffs from the same donors indicate that iTreg are intermediate between expanded CD4^+^CD25^hi^ tTregs and Teffs, whereas modulation of suppressive activities by PKC-θ and Dlgh1 signaling pathways are shared.

## Introduction

Regulatory Tregs (Tregs) are potentially of great value for therapy in settings where immunological homeostasis is disrupted such as graft-versus-host disease (GVHD)^1^. There are two major subsets of Tregs *in vivo*: thymus-derived, CD4^+^ T cells that express high levels of CD25 marker and Foxp3 transcription factor (tTreg), and peripheral CD4^+^CD25^+^ cells that are induced *in vivo* from CD25^−^ precursors in peripheral tissues (pTregs) and may have less stable expression of Foxp3^2–5^. For clinical GVHD studies, human Tregs have been purified from peripheral blood^6^ or purified and expanded from umbilical cord blood^7^. In adult humans, peripheral blood Treg are a mixture of tTreg and pTreg, whereas the Tregs from cord blood are highly enriched for tTreg presumably due to the relatively low levels of exposure to environmental antigens and microbes that are major antigen sources for driving pTreg generation^5^. The infusion of freshly isolated peripheral blood Tregs or *ex vivo* expanded cord blood Tregs significantly reduced GVHD incidence and severity in patients undergoing allogeneic hematopoietic cell transplantation^6, 8, 9^. However, both sources of freshly isolated Tregs provide a fixed and limited Treg cell number. The generation of *in vitro* induced Tregs (iTregs) from CD4^+^CD25^−^ T cells isolated from adult peripheral blood can yield large number of cells since the frequency of CD4^+^25^−^ T cells is far higher than CD4^+^25^br^ Tregs^10^. Moreover, recent evidence suggests that repertoire differences between tTreg and iTregs provide an opportunity for combinatorial therapy to prevent or treat autoimmune disease^11^. For these reasons, we have initiated an ongoing clinical trial of iTreg infusion to prevent GVHD in patients undergoing allogeneic hematopoietic cell transplantation (M. MacMillan NCT01634217). As such, it is important to determine if intracellular molecular mechanisms that signal suppression are different between human expanded tTregs and iTregs for basic understanding and enhanced utility.

Treg-suppressive function depends on T-cell receptor (TCR) signaling and IL-2^12^. Positive and negative signals downstream of the TCR that control Tregs appear to be divergent from conventional effector T cells (Teff) particularly at the level of the immunological synapse (IS), a structured interface between T cells and antigen-presenting cells (APCs)^13, 14^. The activity of human Tregs is restrained by Protein Kinase C-θ (PKC-θ)^13^. PKC-θ is recruited to the IS and essential for optimal Teff activation^15–19^, but is not recruited to the IS in freshly isolated from human peripheral blood Treg or expanded cord blood tTregs^20^. Moreover, inhibition of PKC-θ activity protects Treg from negative pro-inflammatory signals and restores suppressive function of Tregs from rheumatoid arthritis patients^20, 21^.

In addition to the negative regulatory effects of PKC-θ on Treg suppressor function, the scaffold protein Disc large homolog 1 (Dlgh1) facilitates Treg suppressive function. Both human peripheral blood Treg and cord-blood derived expanded tTreg recruit fourfold more Dlgh1 to the IS compared to Teff^20^. Dlgh1 operates independently of the negative feedback pathway mediated by PKC-θ and related adapter protein Carma1 in Tregs^22, 23^, providing a link between the TCR signaling and stimulation of Treg function^24^.

In this study we investigated localization of signaling molecules Dlgh1 and PKC-θ in the IS of human expanded tTregs, iTregs and Teff cells and determined how interfering with these pathways alters suppressive function of these distinct cell types *in vitro*.

## Results and Discussion

**Figure 1:**
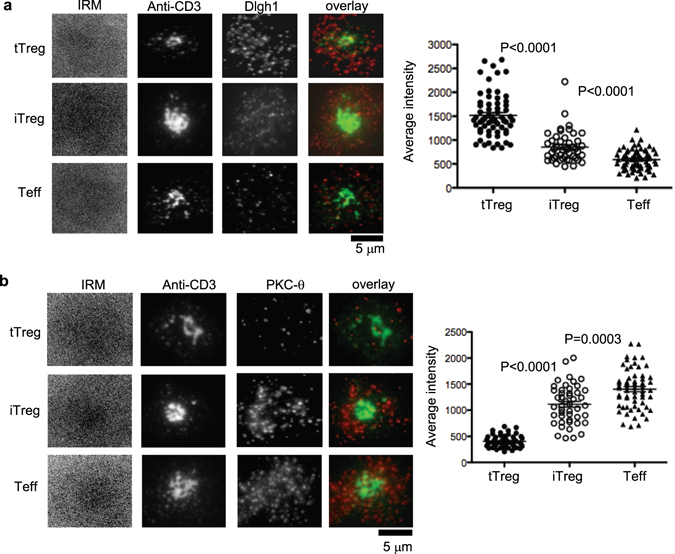
Dlgh1 and PKC-θ are recruited differently to IS in tTregs, iTregs and Teffs. MACS bead purified human CD4^+^CD25^hi^ (tTreg), CD4^+^CD25^−^ T cells activated by TGF-β and Rapamycin (iTregs) and CD4^+^CD25^−^ T cells (Teffs) were introduced into bilayers containing both anti-CD3 (5 µg/ml) and ICAM-1 at 250 molecules/mm^2^ fixed at 20 min and permeabilized, stained with anti-Dlgh1 (**a**) and anti-PKC-θ (**b**) antibodies and imaged by TIRFM. The panels show representative images. Dlgh1 and PKC-θ staining was quantified by calculation of average fluorescence intensity in cells. Data are representative of two different experiments. *P* values were calculated using Mann-Whitney test.

We first sought to compare TCR-induced signaling events in human iTregs, expanded tTreg and Teff cells that were 99%, 88% and 48% respectively double positive for FOXP3 and CD25 markers (Supplementary Figure 1). Levels of Dlgh1 and PKC-θ at the IS were determined by using planar bilayers containing laterally mobile, fluorescently labeled ICAM-1 and stimulatory anti-CD3 antibodies by total internal reflection fluorescence microscopy (TIRFM), which only detects fluorescence within 200 nm of the interface between the T cells and the planar bilayers^20, 25^. The iTreg were prepared by negative selection of CD4^+^CD25^−^ cells from cord blood that was then activated with anti-CD3/anti-CD28 mAbs and expanded with TGF-β, IL-2 and rapamycin^10^. For comparison, cultured Teff were activated with anti-CD3/anti-CD28 mAbs and cultured with IL-2 alone^10^. The tTreg, which served as a positive control for suppressive Treg cells, were first isolated using anti-CD25 coated magnetic nanoparticles, activated with anti-CD3/anti-CD28 mAb and expanded with IL-2^8^. All three T cell subsets were purified from the same donors with at least 3 donors for each experiment. Cells were fixed at 15 minutes and permeabilized prior to staining with antibodies to Dlgh1 or PKC-θ. The iTreg subset had twofold higher Dlgh1 signal in the IS compared to Teff, but a twofold lower signal than that for tTreg cells (Fig. 1a). As expected, Teff displayed the highest level of PKC-θ recruitment to the IS and iTreg displayed a significant 25% reduction in PKC-θ recruitment to the IS; tTreg, display an even more profound, 75% reduction of PKC−θ from the IS compared to Teff (Fig. 1b).

**Figure 2:**
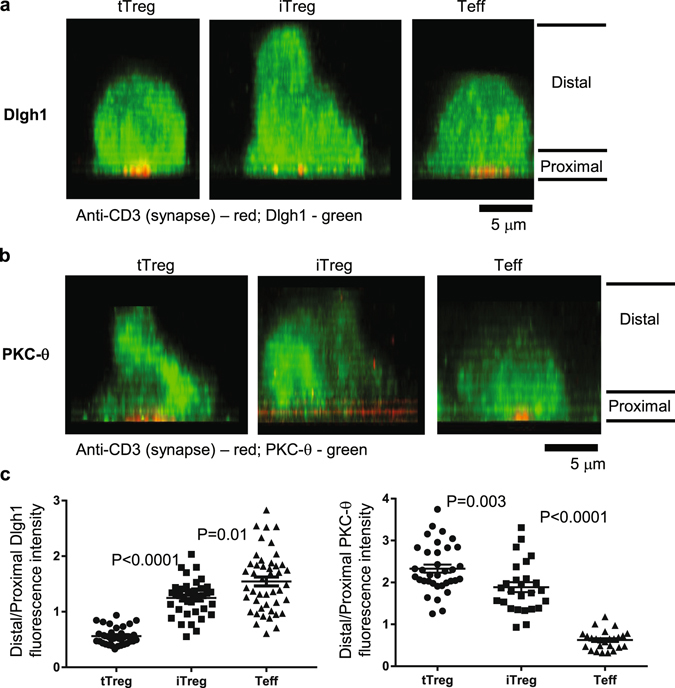
Side view of Dlgh1 and PKC-θ intracellular distribution in tTreg, iTreg and Teffs. The cells were activated using lipid bilayers as mentioned above, for 10 min at 37 °C, fixed and processed for Dlgh1 and PKC-θ immunofluorescence. Cells were imaged using line scanning confocal microscope, and images were 3D projected to reveal intracellular distribution of aforementioned proteins. Anti-CD3 (pseudocolored red) marks synaptic and proximal areas, while the proteins have been pseudocolored green. Note the enriched distribution of Dlgh1 at the proximal and of PKC-θ towards distal side in tTreg (left panels), and a reduction of this distribution in iTreg (middle panels). The panels show representative images. Dlgh1 and PKC-θ staining was quantified by calculation of average fluorescence intensity in cells. Data are representative of two different experiments. *P* values were calculated using Mann-Whitney test.

One mechanism by which PKC-θ is excluded from the IS is based on competition of CTLA-4 with CD28 for binding to CD80 and CD86 on APCs. CD28 recruits PKC-θ, whereas CTLA-4 recruits PKC-ε with different functional consequences^19^. The TCR-dependent, CD28-independent recruitment of PKC-θ to the IS is highly active in Teff^18^, but not in pTreg or tTreg. Indeed, when the whole cell distribution of a Dlgh-1 and PKC-θ was visualized using confocal microscopy, these proteins exhibited opposite patterns of enrichment in Treg and Teff, further emphasizing the intermediate nature of the iTreg based upon the distribution of these proteins compared to tTreg and Teff cells (Fig. 2a and b).

**Figure 3:**
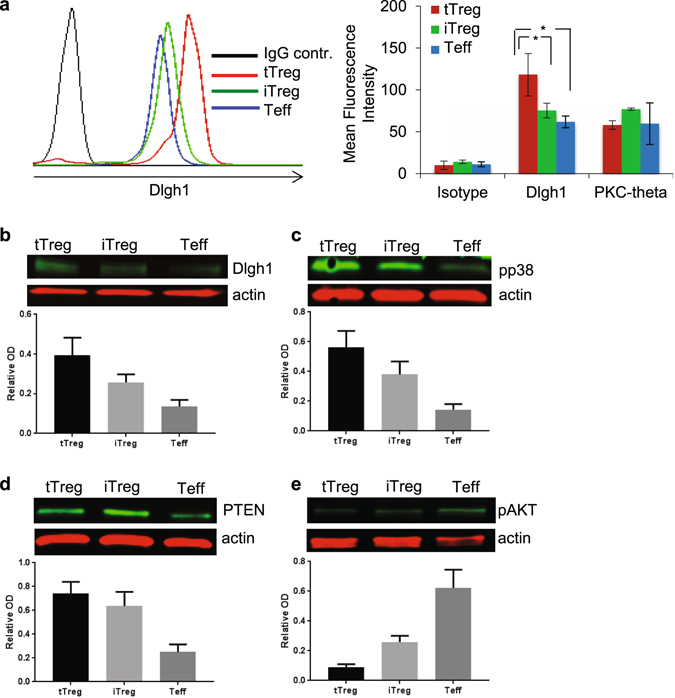
Intracellular levels of Dlgh1 and PKC-θ were determined by intracellular staining by FACS (**a**) or by Western Blot (**b**) in tTregs, iTregs and Teffs. Cells were activated by immobilized anti-CD3 antibodies (5 μg/ml) and lysed. Phosphorylation of p38 (**c**) and AKT (**d**) as well as PTEN protein levels (**e**) was determined by Western Blot. Data are representative of 3 different experiments, the bar charts (B-E) are means ± SEM. *P < 0.05 was calculated by *t* test. Full-length gels are included in Supplementary Figure 2.

To investigate the role of Dlgh1 and PKC-θ pathways in regulation of iTreg function, we evaluated their intracellular levels. Both flow cytometry and immunoblotting on proteins separated by SDS-PAGE revealed that protein levels of Dlgh1 were significantly higher in tTreg compared to iTreg and Teff cells expanded *in vitro* (Figs 3a and 2b, Supplementary Figure 2a), while no differences were observed in PKC-θ levels. We also confirmed our previous report^25^ that Dlgh1 is expressed equally in freshly purified, non-expanded CD4^+^CD25^+^ and CD4^+^CD25^−^ T cells (data not shown). These data suggest that *in vitro* expansion may actually enhance the suppressive signaling capacity of tTreg through up-regulation of Dlgh1.

Dlgh1 selectively recruits p38 leading to activation of Nuclear Factor of Activated T cells (NFAT) in antigen-experienced T cells and freshly purified Tregs^25, 26^. In expanded cells, we found that protein levels of Dlgh1 correlate with the ability of tTreg, iTreg and Teff cells to phosphorylate p38 in response to TCR stimulation (Fig. 3c, Supplementary Figure 2b). It was recently reported that Dlgh1 stabilizes and recruits PTEN to suppress Akt activation in Tregs and other cells^25, 27^. Here, we found that PTEN levels are higher in both tTregs and iTregs compared to Teffs (Fig. 3d, Supplementary Figure 2c) while both types of Tregs have reduced Akt phosphorylation on Ser-473 in response to TCR stimulation (Fig. 3e, Supplementary Figure 2d). This is in agreement with previously published work demonstrating that optimal Treg function requires reduced Akt activation^14, 28^. Thus, regardless of their origin, tTregs and iTregs share common TCR-dependent signaling features, such as increased p38 and diminished AKT phosphorylation compared to Teff cells.

**Figure 4:**
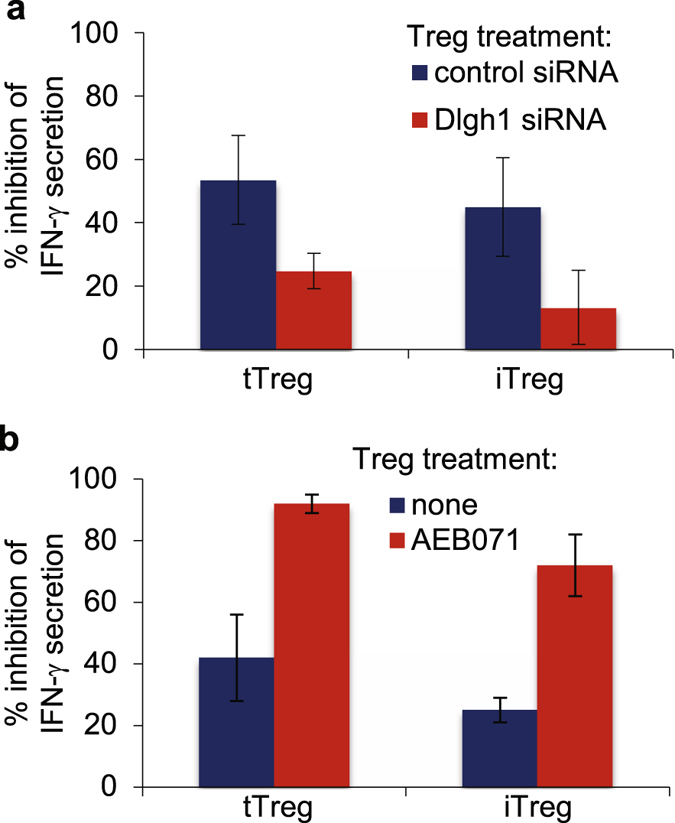
The suppressive function of both tTregs and iTregs is concurrently regulated via Dlgh1/PKC-θ signaling pathways. Treg subsets, tTregs and iTregs were transfected with small interfering RNA (siRNA) targeting Dlgh1 or with control siRNA by AMAXA and plated in presence of IL-2 (300 IU/ml) (**a**) or pre-treated with PKC-θ inhibitor (AEB071) at 10 μM for 30 min and washed three times (**b**). SiRNA-transfected (**a**) or AEB071-treated (**b**) Tregs were mixed with CD4^+^ CD25^−^ T cells at 1:3 ratio and plated on immobilized anti-CD3 antibodies (5 μg/ml). The supernatants were analyzed for IFN-γ secretion after 48 hours by ELISA. Data are representative of two different experiments.

To investigate whether Dlgh1 and PKC-θ pathways are actively involved in regulation of iTreg suppressive function, we specifically silenced Dlgh1 gene expression using RNA interference (RNAi) in both tTregs and iTregs. Treatment with a mixture of four specific siRNAs for Dlgh1 resulted in an 87% and 88% reduction of Dlgh1 expression in tTregs and iTregs respectively (Supplementary Figure 3a). This reduction of Dlgh1 significantly impaired the ability of both types of Tregs to suppress IFN-γ (Fig. 4a) and IL-17 (Supplementary Figure 3b) secretion in CD4^+^CD25^−^ T cells. Dlgh1 silencing also reduced PTEN protein levels in iTregs (Supplementary Figure 3c) to the similar extent as was recently shown in freshly purified Tregs^25^. Interestingly, 50% reduction of PTEN in individuals with PTEN hamartoma tumor syndrome appears to be compensated to maintain normal Treg function, despite the patients often suffering from autoimmunity^29^. In contrast, we found that acute reduction of PTEN by >50% secondary to Dlgh1 knock down resulted in a profound functional defect. Conversely, pharmacological inhibition of PKC-θ significantly up-regulated the suppressive function of both expanded tTregs and iTregs (Fig. 4b), while completely blocking CD4^+^CD25^−^ T cell function as measured by IFN-γ secretion (Supplementary Figure 3d). In addition, pretreatment of both types of Tregs, but not of Teffs with PKC-θ inhibitor significantly up-regulated their ability to suppress target cell proliferation in the co-culture experiment (Supplementary Figure 3e). Thus, consistent with comparable intracellular molecular signatures both tTregs and iTregs are concurrently regulated via Dlgh1 and PKC-θ signaling pathways.

In conclusion, direct expansion of cord blood-derived tTreg or induction of iTreg demonstrates a requirement for Dlgh1 in suppressor function and a restraint on suppressive function through the PKC-θ signaling pathway. The iTreg cells appear to share similar functional requirements and responses to both Dlgh1 knockdown and interruption of PKC-θ signaling with tTregs, although the distribution of the signaling proteins was less extremely distinct from Teff compared to freshly purified peripheral blood Tregs (pTreg/tTreg) or cord blood derived expanded Tregs (tTreg). While neuropilin-1 has been used as a marker for murine tTreg, there is no accepted surface marker to distinguish human tTreg and pTreg^2, 5^. In this context, it’s clear that mouse pTreg have an intermediate level of chromatin remodeling in the FoxP3 locus compared to tTreg and Teff^30^, which is thought to influence functional stability more than effector function. It is interesting to consider and further study whether the more distinctive IS features of tTreg compared to iTreg may contribute to greater suppressor cell functional stability. This could be a consideration in therapeutic applications and efforts to optimize iTreg performance. Nonetheless, iTreg have fully re-tasked Dlgh1 and PKC−θ signaling compared to Teff cells, but the degree to which the IS is reorganized appears intermediate state between tTregs and Teffs with regard to the distribution of Dlgh1 and PKC-θ at the IS. Biochemical analysis of these three subsets revealed that TCR signaling leads to increased levels of p38 phosphorylation and PTEN expression in both tTregs and iTregs followed by reduced levels of Akt phosphorylation. These data suggest that regardless of their origin, Dlgh1 is essential for optimal Treg function by scaffolding two critical signaling pathways to generate a defined signaling milieu, in which p38 activation is high and Akt activation is low through a positive regulation of PTEN. Finally, the suppressive function of both tTregs and iTregs can be potentially manipulated through Dlgh1 and PKC-θ pathways. Thus, in addition to provide insights into a basic understanding of molecular differences between tTregs and iTregs our present work can be valuable in designing effective Treg-based immunotherapies to treat autoimmunity and graft versus host disease in clinic^7^.

## Material and Methods

### Cell purification

Cord blood CD4^+^ CD25^+^ (tTregs) and CD4^+^ CD25^−^ (Teff) T cells were isolated from frozen UCB units by positive selection using directly conjugated anti-CD25 magnetic microbeads and expanded as previously described^8, 31^. UCB units used for this study were collected under approved IRB protocol under the American Red Cross Cord Blood Program (through Dr. D. McKenna, University of Minnesota) and informed consent was obtained from all subjects. All methods were carried out in accordance with relevant guidance and regulations. Cells were cultured with anti-CD3/CD28 mAb-coated Dynabeads (3:1 bead/cell) for 18 to 21 days and split every 2 to 3 days. Recombinant IL-2 (300 IU/ml; Chiron, Emeryville, CA) was added on either day 0 (Teff and iTreg) or day 3 (tTreg) and maintained for culture duration. iTregs were induced by culturing CD25^−^CD4^+^ T cells in the presence of TGF-β (10 ng/ml) and Rapamycin (109 nM) in addition to CD3/28 mAb-coated beads and IL-2^10^.

### Planar lipid bilayers

Planar lipid bilayers containing anti-CD3 antibodies (5 μg/ml) and ICAM-1 (250 molecules/mm^2^) were prepared in parallel-plate flow cells as described previously^20^. The flow cell containing the bilayers was warmed up to 37 °C, cells were injected in 500 ml of HEPES-buffered saline containing 1% human serum albumin, and images were collected on a custom automated Nikon inverted fluorescence microscope.

### Microscopy

All TIRF imaging was performed on the custom automated Nikon inverted fluorescence microscope using the 100X/1.45 N.A. TIRF objective from Nikon. TIRF illumination was set up and aligned according to the manufacturer’s instructions as previously described^32^. Briefly, cells interacted with the bilayers for 20 min at 37 °C, fixed with 2% PFA, permeabilized with 0.05% Triton-X 100, blocked and stained with rabbit polyclonal antibodies to Dlgh1 (H-60; sc-25661) or PKC-θ (sc-212) from Santa Cruz Biotech (CA) for 20 min, and then incubated with fluorescently tagged goat anti-rabbit Fab_2_ (Invitrogen, Carlsbad, CA). Controls included the use of nonimmune species-matched IgG. Measurement of signaling was done as previously described^20^. Confocal microscopy was carried out on a Zeiss LSM 510 Meta imaging system (63 × 1.4 NA; Zeiss, Jena, Germany) using appropriate factory-set filters and dichroics for different fluorophores as previously described^20^.

### Flow cytometry

For intracellular staining, indicated populations of T cells were fixed and permeabilizied with Fixation/Permeabilization buffer set (00–5523; eBioscience), washed, and stained (30 min, 4 °C) with primary antibodies (PKC-θ or Dlgh1). Then, the cells were incubated (30 min, 4 °C) with a FITC-conjugated secondary antibodies (Jackson ImmunoResearch Lab. Inc., West Grove, PA). Human-specific antibodies used for flow cytometry CD4 (clone RPA-T4) and CD25 (M-A251), were purchased from eBioscience, while FOXP3 (clone 249D) is from BioLegend. Cells were stained for CD4, CD25, FOXP3 using the BioLegend FOXP3 intracellular staining kit. We analyzed samples in a FACSCalibur machine (BD, Franklin Lakes, NJ).

### RNA interference

SiRNA duplexes (siRNAs) were synthesized and purified by Qiagen Inc (Valencia, CA) as described^33^. Mixture of four Dlgh1 specific siRNAs was used (catalog numbers SI00059584 (Dlgh1–1), SI02632518 (Dlgh1–7), SI03046099 (Dlgh1–8) and SI03102799 (Dlgh1–9)). Control siRNA was purchased from Qiagen (1027281). Transfections of human T cells were performed using the human T cell Nucleofector kit (Amaxa Biosystems, Lonza, Basel, Switzerland) and protocol for activated primary human T cells as previously described^20^.

### Western Blot analysis

Cells were lysed in RIPA buffer supplemented with protease and phosphatase inhibitors for 20 min at 4 C. After 20 min centrifugation at 10000 g cell lysates were loaded on an SDS-PAGE gel and transferred to nitrocellulose membrane. The membranes were blocked, probed with the specific antibodies overnight, washed, and stained with secondary antibodies from Li-Cor, Inc. (Lincoln, NE) Immunoreactive protein bands were visualized using an Odyssey Infrared Imaging system. Anti-alpha actin antibodies were used as loading controls.

### *In vitro* suppression assays

Different subsets of Tregs were treated or not, washed three times, and added at ratio 1:3 (1.25 × 10^5^ Tregs: 5 × 10^5^ CD4^+^CD25^−^ T cells) to CD4^+^CD25^−^ T cells at final concentration 2 × 10^6^/ml. The cells were co-cultured on anti-CD3 mAb (5 μg/ml) pre-coated 24-well plates for 48 hr. The PKC-θ inhibitor, AEB071, was provided by Novartis (Ridgefield, CT) and dissolved in DMSO. T cells were pretreated for 30 min with 10 μM at 37 °C and washed three times. Cytokine secretion was determined by ELISA as previously described^20^, using Human IL-17 (R&D Systems Inc, Minneapolis, MN) and IFN-γ Cytoset^tm^ (Biosource; Camarillo, CA). The capacity of expanded Tregs to suppress target cell proliferation *in vitro* was assessed by using 5-carboxyfluorescein-diacetate-succinimide ester (CFSE) inhibition assay^31^. Peripheral blood mononuclear cells (PBMCs) were purified, labeled with CFSE (InVitrogen), and stimulated with anti-CD3 mAb-coated beads (Dynal) in 96-well round-bottom plates with or without expanded cells (Treg:PBMC at ratios of 1:4-1:64). Assays were harvested on day 4. Cells were stained with anti-CD4 and/or −8 mAb and data acquired by LSRII. Data was analyzed using the proliferation platform in FlowJo (Treestar, Ashland, OR), and suppression determined from the Division Index.

### Statistics

We determined P values by Mann-Whitney test or two-tailed t-test by using the GraphPad Prism software (San Diego, CA).

## Electronic supplementary material

Supplementary figures
