# Author Correction: Human in vitro-induced regulatory T cells display Dlgh1 dependent and PKC-θ restrained suppressive activity

**DOI:** 10.1038/s41598-020-60485-6

**Published:** 2020-02-19

**Authors:** Alexandra Zanin-Zhorov, Sudha Kumari, Keli L. Hippen, Sarah C. Merkel, Margaret L. MacMillan, Bruce R. Blazar, Michael L. Dustin

**Affiliations:** 1 Molecular Pathogenesis Program, Skirball Institute of Biomolecular Medicine, Department of Pathology, New York University School of Medicine, New York, NY 10016 USA; 2 University of Minnesota Cancer Center and Department of Pediatrics, Division of Blood and Marrow Transplantation, Minneapolis, MN 55455 USA; 3 Kadmon Corporation, LLC, New York, NY 10016 USA; 4 Koch institute of Integrative Cancer Research, MIT, Cambridge, MA 02139 USA; 5 Kennedy Institute of Rheumatology, University of Oxford, Oxford, OX3 7FY UK

Correction to: *Scientific Reports* 10.1038/s41598-017-04053-5, published online 26 June 2017

The original version of this Article contained an error in the title of the paper, where “Dlgh1 dependent” was incorrectly given as “Dlgh1dependent”. This has now been corrected in the PDF and HTML versions of the Article and in the accompanying Supplementary figures file.

