# Supplementary material for: Human in vitro-induced regulatory T cells display Dlgh1 dependent and PKC-θ restrained suppressive activity
Source: Sci Rep. 2017 Jun 26;7:4258. doi: 10.1038/s41598-017-04053-5 (PMC5484704; doi:10.1038/s41598-017-04053-5)
Supplement: Supplementary file 1 — Supplementary figures [file 41598_2017_4053_MOESM1_ESM.pdf]

Human in vitro-induced regulatory T cells display Dlgh1 dependent and PKC-θ restrained suppressive activity

Alexandra Zanin-Zhorov[1,3*], Sudha Kumari[1,4], Keli L. Hippen[2], Sarah C. Merkel[2], Margaret L. MacMillan[2], Bruce R. Blazar[2], and Michael L. Dustin[1,5*]

[1]Molecular Pathogenesis Program, Skirball Institute of Biomolecular Medicine, Department of Pathology, New York University School of Medicine, New York, NY10016, USA

[2]University of Minnesota Cancer Center and Department of Pediatrics, Division of Blood and Marrow Transplantation, Minneapolis, MN 55455, USA

[3]Current address is Kadmon Corporation, LLC, New York, NY 10016, USA

[4]Current address is Koch institute of Integrative Cancer Research, MIT, Cambridge, MA-02139, USA

[5]Current address is Kennedy Institute of Rheumatology, University of Oxford, Oxford OX3 7FY, UK

[*]Corresponding authors: Alexandra Zanin-Zhorov PhD: Alexandra.Zanin-Zhorov@kadmon.com

Michael L Dustin PhD: michael.dustin@kennedy.ox.ac.uk

Running title: Dlgh1 and PKC-θ oppositely controls in vitro-induced human Tregs

Characteristics of expanded T cell subsets:

a

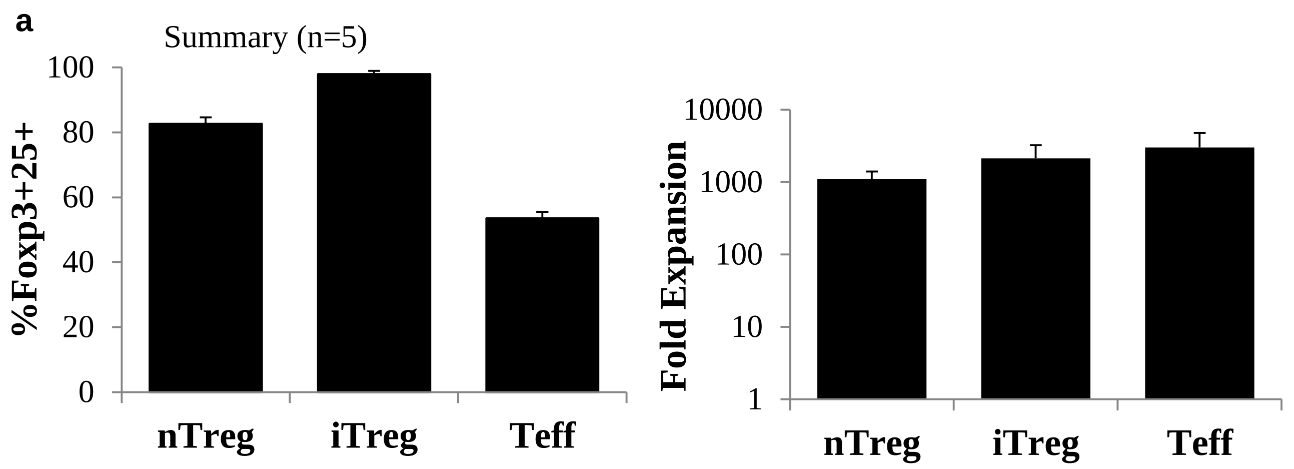

b

Representative FACS plot examples

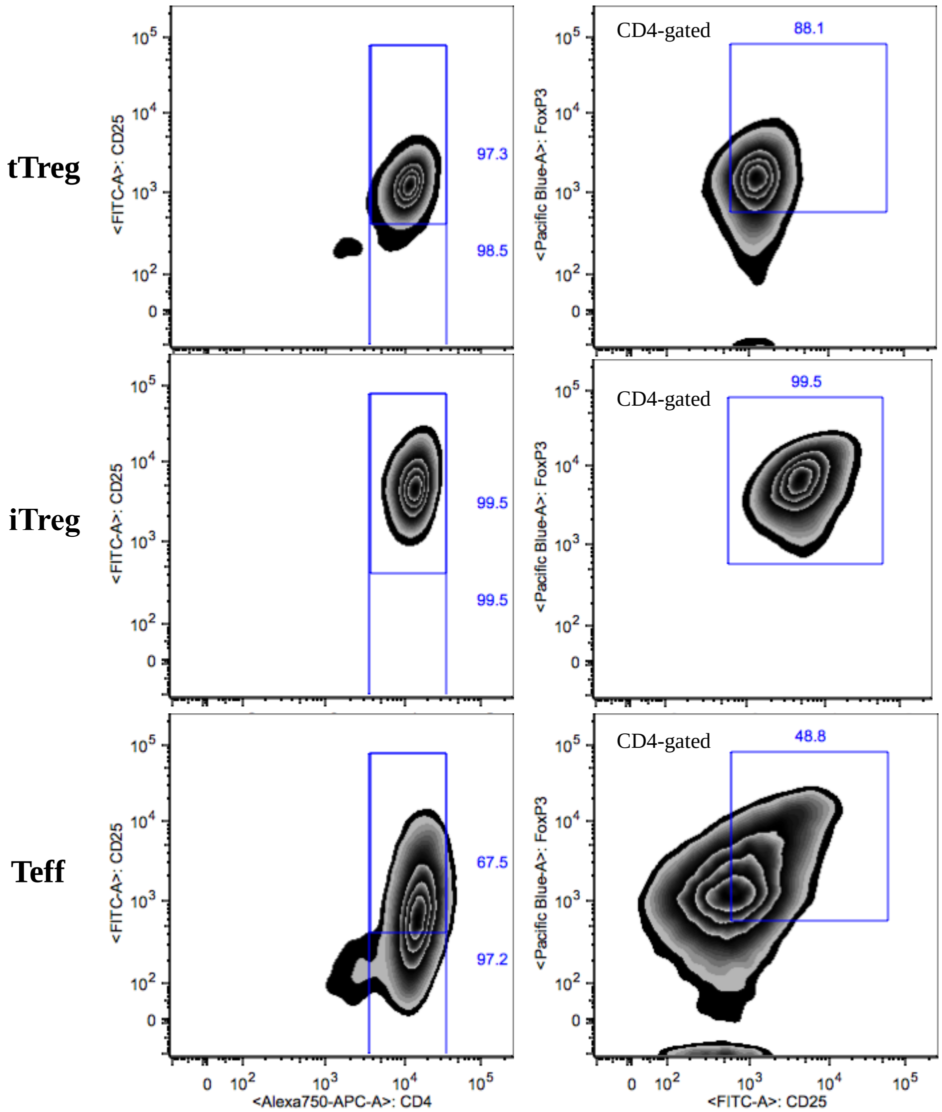

Supplementary Figure 2

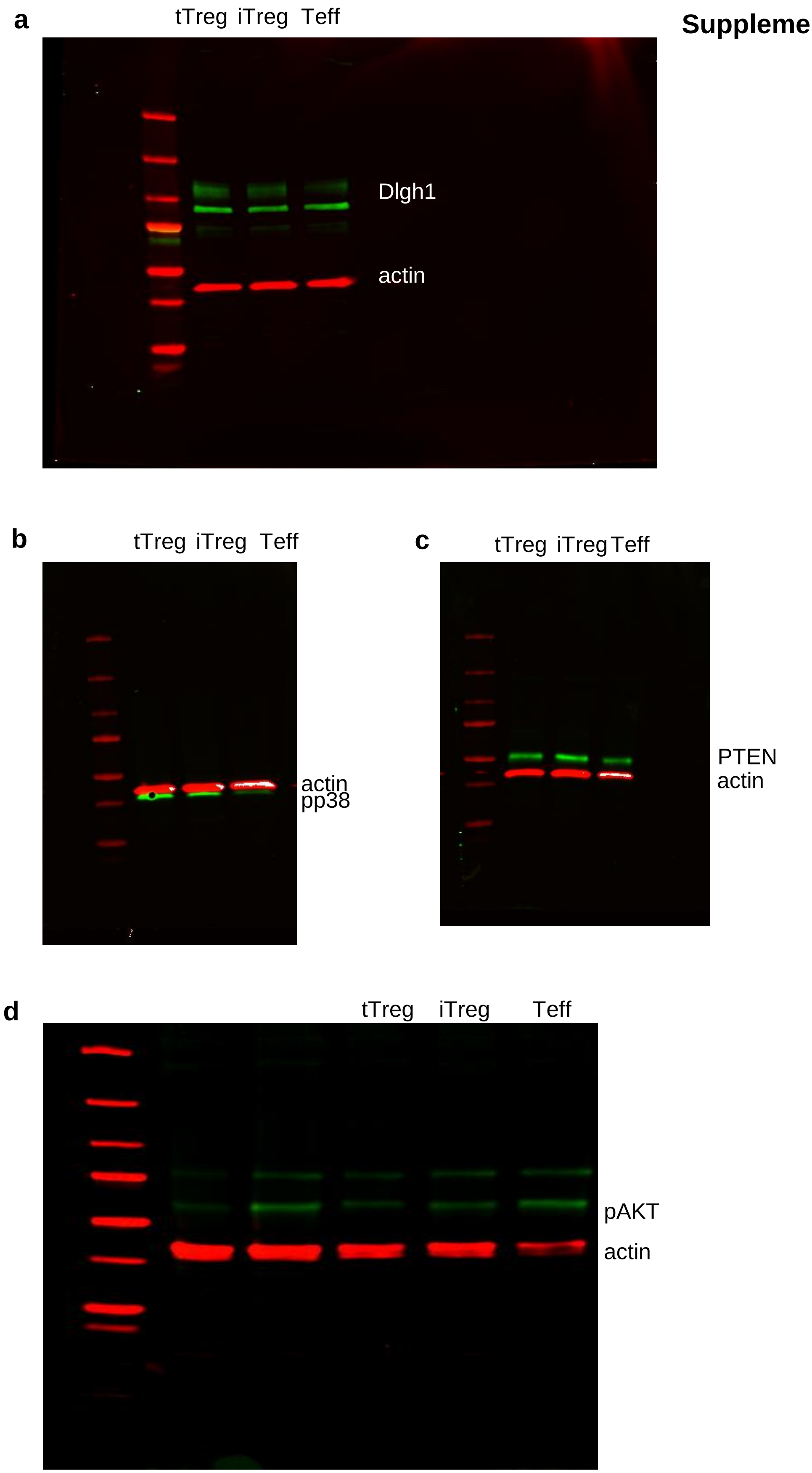

Supplementary Figure 3

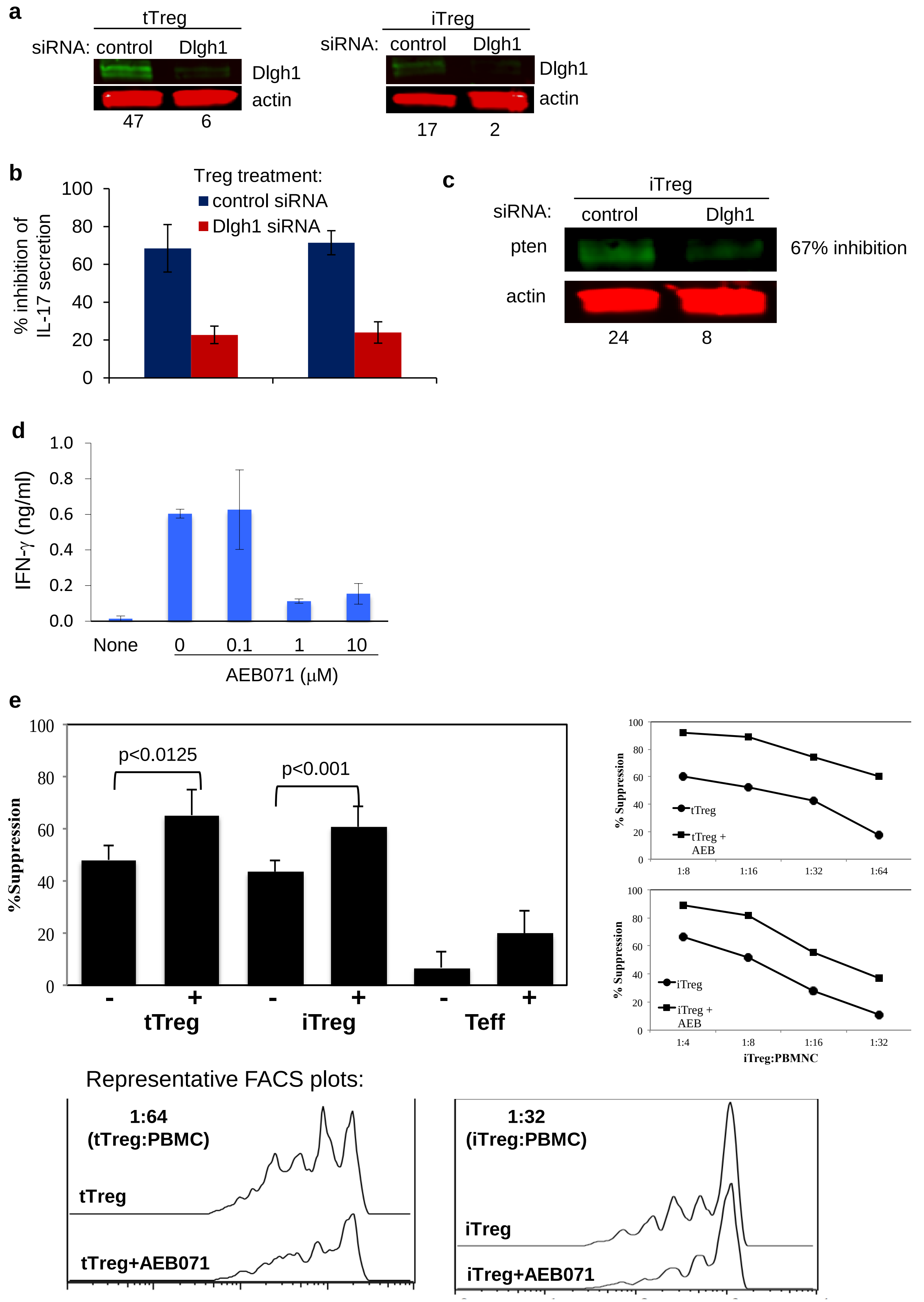

Supplementary figure legend

Figure 1. tTreg, iTreg, and Teff cells were purified from UCB and expanded in vitro. Summary of $CD25^{+}Foxp3^{+}$ purity and average fold expansion for the indicated cell types on day 14 of culture (a). Representative examples of CD4, CD25 and Foxp3 staining for the cultured cells are shown (b).

Figure 2. Full-length gels for Figure 3b-e.

Figure 3. Dlgh1 and PKC-θ oppositely regulates iTregs. Treg subsets, tTregs and iTregs were transfected with small interfering RNA (siRNA) targeting Dlgh1 or with control siRNA by AMAXA and plated in presence of IL-2 (300 IU/ml). After 48 hours Dlgh1 (a) and PTEN (c) expression was measured by Western blot analysis. SiRNA-transfected Tregs were mixed with $CD4^{+}$ $CD25^{-}$ T cells at 1:3 ratio and plated on immobilized anti-CD3 antibodies (5 μg/ml). The supernatants were analyzed for IL-17 secretion after 48 hours by ELISA (b). $CD4^{+}$ $CD25^{-}$ T cells were treated with indicated concentrations of PKC-θ inhibitor (AEB071) and plated on immobilized anti-CD3 mAb. The supernatants were analyzed for IFN-γ after 48hours by ELISA (d). In vitro expanded tTregs, iTregs and Teffs were treated ± AEB071 (10μM) for 30 minutes, washed, and then assayed for suppressive function using a proliferation assay in which cultured cells are incubated with CFSE-loaded allogeneic PBMC at doses from 1:4 to 1:64 (expanded cell:PBMC) and stimulated with anti-CD3 beads for 4 days. Since allo-PBMC are from different donors for each assay, overall suppression varies enough such that no single ratio of (expanded cell:PBMC) was useful for all experiments. Therefore, the ratio at which untreated Treg suppression was closest to 50% was chosen for analysis in each experiment (varied from 1:8 to 1:32). Data represent a summary of 4-8 independent experiments.
